# A Hybrid Chatbot to Promote Pneumococcal Vaccination Among Older Adults: A Randomized Clinical Trial

**DOI:** 10.1001/jamanetworkopen.2025.35813

**Published:** 2025-10-08

**Authors:** Zixin Wang, Siyu Chen, Josiah Poon, Soyeon Caren Han, Danhua Ye, Fuk-yuen Yu, Yuan Fang, Zhao Ni, Martin C. S. Wong, Phoenix K. H. Mo

**Affiliations:** 1 Centre for Health Behaviours Research, JC School of Public Health and Primary Care, the Chinese University of Hong Kong, Hong Kong SAR, China; 2 School of Computer Science, the University of Sydney, Sydney, New South Wales, Australia; 3 School of Computing and Information System, the University of Melbourne, Melbourne, Victoria, Australia; 4 Department of Health and Physical Education, the Education University of Hong Kong, Hong Kong SAR, China; 5 School of Nursing, Yale University, New Haven, Connecticut; 6 Centre for Interdisciplinary Research on AIDS (CIRA), Yale University, New Haven, Connecticut; 7 Centre for Health Education and Health Promotion, JC School of Public Health and Primary Care, the Chinese University of Hong Kong, Hong Kong SAR, China

## Abstract

**Importance:**

There are few robust evaluations assessing the efficacy of chatbots to improve pneumococcal vaccination (PV) uptake among adults 65 years of age or older.

**Objective:**

To evaluate the relative efficacy of a hybrid chatbot in increasing PV uptake among Hong Kong residents aged 65 years or older.

**Design, Setting, and Participants:**

This partially masked, parallel-group randomized clinical trial was conducted between May 1, 2023, and November 30, 2024 in Hong Kong, China. Participants were aged 65 years or older, had a Hong Kong identity card, could speak and comprehend Cantonese, were smartphone and WhatsApp users, and had no prior PV uptake. Participants were recruited through random telephone calls and were randomized to either the stage of change group or the standard intervention group.

**Interventions:**

In the stage of change group, the rule-based component of the hybrid chatbot assessed participants’ stage of change regarding PV uptake and then delivered stage of change–tailored interventions at months 0, 1, 2, and 3. The natural language processing component of the hybrid chatbot provided real-time answers to participants’ PV-related questions. In the standard intervention group, the chatbot sent participants a link to access a standard online video covering PV information at months 0, 1, 2, and 3.

**Main Outcomes and Measures:**

The primary outcome was self-reported PV uptake at month 12, which was validated by the research team. The secondary outcome was participants’ stage of change measured at month 0 and month 12 by using validated questions, with a score of 1 = precontemplation, 2 = contemplation, 3 = preparation, and 4 = action.

**Results:**

A total of 374 participants (213 female [57.0%]; mean [SD] age, 69.6 [3.1] years) were randomized to either the stage of change group (n = 187) or the standard intervention group (n = 187). The intention-to-treat analysis showed that the validated PV uptake rate was higher in the stage of change group than in the standard intervention group (29.4% vs 18.7%; *P* = .01). The mean (SD) stage of change score was higher in the stage of change group than in the standard intervention group (2.2 [1.3] vs 1.9 [1.1]; *P* = .02). More participants in the stage of change group than in the standard intervention group completed at least 1 intervention session (79.7% vs 57.8%; *P* < .001).

**Conclusions and Relevance:**

In this randomized clinical trial, the hybrid chatbot was more efficacious than the standard intervention in increasing PV uptake among older adults in Hong Kong. A hybrid chatbot may be a sustainable PV promotion for older adults.

**Trial Registration:**

ClinicalTrials.gov Identifier: NCT05772117

## Key Points

**Question:**

Can a hybrid chatbot combining a rule-based component delivering co-designed tailored interventions and an artificial intelligence component providing natural language processing–supported real-time answers to participants’ questions increase pneumococcal vaccination (PV) uptake among older adults?

**Findings:**

In this randomized clinical trial of 374 Hong Kong residents 65 years of age or older, those receiving the hybrid chatbot intervention at months 0, 1, 2, and 3 had significantly higher PV uptake than those receiving a chatbot-delivered standard online intervention without real-time answers to questions.

**Meaning:**

Findings from this trial indicated that a hybrid chatbot may serve as a sustainable strategy to promote PV uptake among older adults.

## Teaser

This randomized clinical trial compares the relative efficacy of a hybrid chatbot delivering tailored interventions and providing real-time answers with a chatbot delivering the standard intervention to increase pneumococcal vaccination uptake among adults 65 years of age or older.

## Introduction

People 65 years of age or older in Hong Kong have higher risk of pneumococcal disease (PD) and invasive PD (IPD) than people younger than 65 years, with an IPD incidence of 4 in 100 000 and a case fatality rate between 21.4% and 40.0%.^1^ Pneumococcal vaccination (PV) is effective in preventing community-acquired PD and IPD among people aged 65 years or older without safety concerns.^2,3^ The Hong Kong Department of Health recommends that people in this age group receive PV,^4^ and free PVs are offered to Hong Kong residents in this age group (eAppendix 1 in Supplement 1).^5^ However, only 35.3% of these individuals received a PV in 2023.^6^

Four randomized clinical trials (RCTs) found that telephone education sessions delivered by volunteers or nurses or home visits by nurses were more effective than the standard-of-care in increasing PV uptake among community-dwelling older adults.^7,8,9,10^ However, these interventions were resource demanding. It is important to develop sustainable PV promotion. Providing interventions that are tailored to one’s characteristics is an effective way to address vaccine hesitancy^11^; however, tailored interventions to promote PV among older adults are lacking.

Chatbots are computerized programs that replicate human interactions through various forms of communication.^12^ Chatbots have demonstrated good abilities to reach the target population and increase vaccination uptake.^13,14^ In the present study, we assessed whether PV uptake would be increased through the use of a hybrid chatbot that combined both a rule-based system and artificial intelligence–powered components. The rule-based part ensured that carefully co-designed interventions were delivered as intended. The artificial intelligence component supported question and answer (Q&A) sessions by leveraging natural language processing. This effective engagement strategy enables chatbots to better understand users’ open-ended questions and to generate real-time answers.^15^ This hybrid combination is novel for a vaccine chatbot.

The co-designed interventions were guided by the stage of change model, one of the most commonly used stage models that postulates that completed behavioral change will go through 5 ordinal stages: precontemplation, contemplation, preparation, action, and maintenance (eAppendix 2 in Supplement 1). The stage of change model suggests that interventions should be tailored to one’s current stage to facilitate sustained behavioral changes.^16^ A meta-analysis supported the effectiveness of stage of change–tailored interventions in increasing vaccination uptake.^17^

The present RCT was conducted to compare the efficacy of a hybrid chatbot (stage of change–tailored interventions with a real-time Q&A function, hereinafter known as the stage of change group) vs a chatbot-delivered standard online intervention without the real-time Q&A function (hereinafter called the standard intervention group) in increasing PV uptake among Hong Kong residents aged 65 years or older. The interventions received by the standard intervention group simulated existing PV promotion in Hong Kong. We used a chatbot to deliver this intervention to balance the effects caused by the chatbot use in the stage of change group. We hypothesized that the stage of change group would have a higher rate of PV uptake during the 12-month follow-up period compared with the standard intervention group.

## Methods

### Study Design and Participants

This partially masked, parallel-group RCT was conducted between May 1, 2023, and November 30, 2024, in Hong Kong. The outcome assessors (D.Y., F.-Y.U.) and data analysts (S.C., Y.F.) were blinded to the participant allocation. The trial protocol and statistical plan are available in Supplement 2. Verbal informed consent was obtained from all participants rather than written consent because the study was conducted via telephone or a messaging and video calling app. The Survey and Behavioral Research Ethics Committee of the Chinese University of Hong Kong and the Joint CUHK-NTEC Clinical Research Ethics Committee approved the study. This report followed the Consolidated Standards of Reporting Trials (CONSORT) guideline.

The participant inclusion criteria included (1) being 65 years of age or older, (2) possessing a Hong Kong identity card, (3) having the ability to speak and comprehend Cantonese, (4) having the free text messaging and video calling app WhatsApp installed on their smartphones, (5) having no history of PV, and (6) having the ability to send and read text and voice messages via a smartphone. Individuals who were blind or deaf, had a known contraindication of PV^18^ or a major psychiatric illness (schizophrenia and bipolar disorder) or dementia, or scored 16 points or lower on the validated telephone version of the Cantonese Mini-Mental State Examination were excluded. The Cantonese Mini-Mental State Examination has a maximum score of 26, with lower scores reflecting greater cognitive impairment; a cutoff of 16 points or lower indicates possible cognitive impairment.^19^

A previous study by members of our group showed that 18.6% of unvaccinated individuals aged 65 years or older intended to receive a free PV in the next year.^20^ For planning purposes, we conservatively assumed that 30% of participants in the standard intervention group would show an intention after exposure to the intervention, and 50% of those with such an intention would receive the PV (15% in the standard intervention group). We used the smallest detectable difference of 15% between the stage of change group and standard intervention group (30% in the stage of change group). Therefore, 121 participants per group were needed to achieve the planned effect sizes and power of 0.8 and α of 0.05. Assuming that the rate of participants unavailable for follow-up would be 35% at month 12, the total sample size needed was 374 (187 per group), calculated using PASS 11.0 (NCSS) software.

Participants were recruited through random telephone calls.^21^ All household telephone numbers listed in the most up-to-date telephone directories (approximately 350 000 records) were input in a spreadsheet file (Excel; Microsoft Corp). A total of 4000 household numbers were then randomly selected. Trained telephone interviewers conducted the telephone calls between 6:00 pm and 10:00 pm on weekdays and between 2:00 pm and 9:00 pm on Saturdays to avoid undersampling of working individuals. If no one in the household answered the initial call, 4 more follow-up calls were made on different days and hours before the household was considered to be nonvalid. If there was more than 1 eligible individual in the same household, the person whose most recent birthday was closest to the interview date was invited to join the study. The interviewers screened prospective participants for eligibility, briefed them about the study, and guaranteed their anonymity and right to quit at any time. These individuals were informed of the available hotline for inquiry during office hours. Verbal informed consent was obtained. The interviewers signed a form pledging that the participants had been fully informed about the study.^21^ A supermarket coupon worth HK$50.00 (US $6.40) was mailed to the participant after completing each telephone survey at baseline (T0) and 12 months after completion of the intervention (T1).

### Randomization, Masking, and Development of the Intervention

At the end of the baseline survey, the interviewers connected participants to the chatbot system. Participants were then evenly assigned to either the stage of change group or the standard intervention group by a randomization algorithm built into the chatbot system. The automated randomization was conducted online, and the research team was blinded to the treatment allocation.

We used a 3-stage co-design approach to develop the intervention materials and the chatbot system (eAppendix 3 in Supplement 1).^22^ Our chatbot was not publicly available; thus, only participants of this study had access to the chatbot during the project period. We integrated the chatbot with the messaging and video calling app platform via its public web application programming interface (eFigure 1 in Supplement 1). To meet the needs of older adults, both videos and texts were used to deliver health communication messages, and users could use flexible input options (clicking a button on screen, using voice messages, texting, or handwriting) to interact with the chatbot. Details of the hybrid chatbot are provided in eAppendix 3 in Supplement 1.

### Stage of Change and Standard Intervention Groups

**Figure 1.**
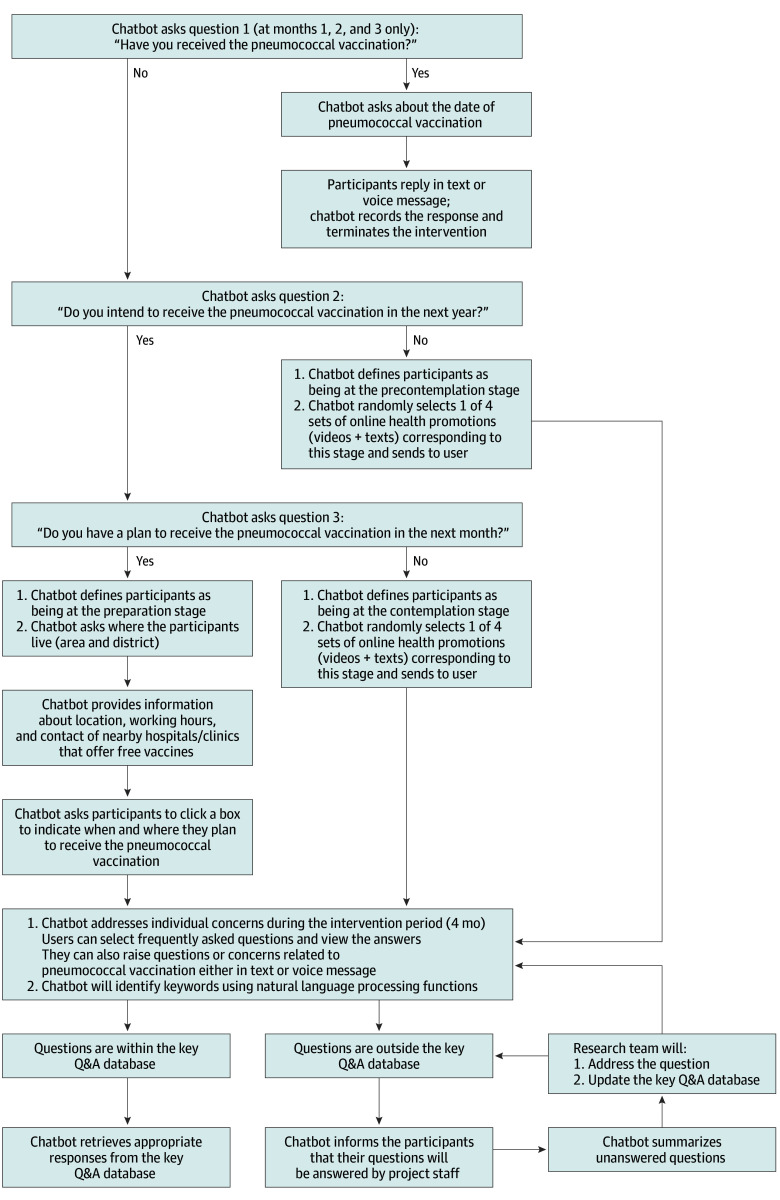
Workflow of the Chatbot in the Stage of Change Group Q&A indicates question and answer.

The stage of change group had access to the hybrid chatbot. The rule-based component first assessed participants’ stage of change regarding PV uptake and then delivered co-designed stage of change–tailored interventions in 4 monthly sessions at month 0, 1, 2, and 3. The Q&A component, supported by a co-created comprehensive Q&A database, allowed users to raise questions and provided real-time answers (Figure 1). The contents of the stage of change–tailored interventions followed the behavioral change strategies recommended by the stage of change and addressed modifiable determinants of PV uptake among older adults residing in Hong Kong.^6^

At the beginning of each session, the chatbot asked 2 questions: whether the participants intended to receive the PV in the next year and whether they planned to do so within the next month. The definitions of the stages of change were as follows: (1) the precontemplation stage, without intention to receive the PV in the next year; (2) contemplation stage, with intention to receive the PV in the next year but without plans to do so in the next month; and (3) preparation stage, having plans to receive the PV within the next month. Starting from the second session, the chatbot asked an additional question to confirm whether the participants had received the PV. If participants selected yes, the chatbot would record the response and then terminate the intervention automatically.

Participants in the precontemplation stage watched an online video that aimed to increase participants’ awareness of the importance of PV. The chatbot randomly selected 1 of 4 different versions of videos in each session to prevent participants from watching the same video twice. In the video, a primary care physician explained the high risk of PD and IPD among older adults in detail. The physician also briefly introduced the notions that PV is effective at protecting older adults and their family members, that local health authorities and physicians strongly recommend older adults receive a PV, and that the PV is available at no cost to older adults.

Participants in the contemplation stage watched a different online video that aimed to increase self-efficacy and perceived benefits and reduce perceived costs related to the PV. In the video, the same physician explained the efficacy and safety of PV in detail. Several vaccinated older adults provided testimonials in the video regarding the mild adverse effects of the PV to reduce the perceived cost of the PV. The physician also encouraged participants to make plans to receive the PV to increase self-efficacy.

For participants in the preparation stage, the chatbot first asked where the participant was living (area and district) and then provided information about location, working hours, and contact information of hospitals offering free PVs for older adults near that area. The participants were also asked to click a box to indicate when and where they planned to receive the PV. This content sought to assist participants in developing and implementing an action plan and to increase their perceived self-efficacy related to PV uptake.

Participants could select 1 or more frequently asked questions related to the PV. The chatbot invited the participants to raise PV-related questions outside the frequently asked questions at each session. The chatbot retrieved relevant answers from the key Q&A database using keyword matching and provided real-time responses in text messages. Participants were free to ask as many questions as they wanted. Questions outside the Q&A database were answered by project staff later through the messaging and video calling app or telephone.

**Figure 2.**
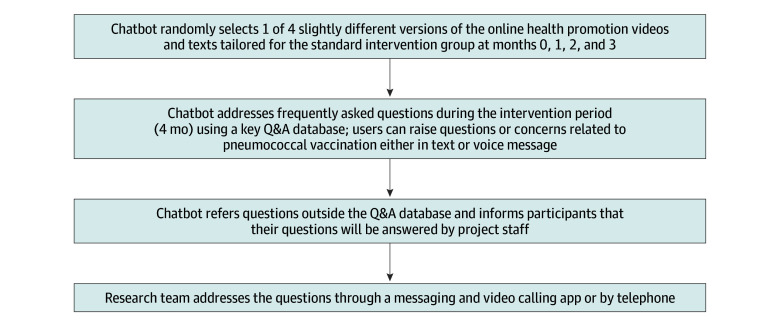
Workflow of the Chatbot in the Standard Intervention Group Q&A indicates question and answer.

In the standard intervention group, the chatbot automatically sent participants a link to access a standard online video presented by the same physician at month 0, 1, 2 and 3 that covered the same key content (ie, high risk of PD and IPD, efficacy and safety of PV, and availability of free PVs) and was the same length (3 minutes) as those for the stage of change group. Such information was identical to that disseminated through mass media channels by the Hong Kong government. Similarly, the chatbot randomly selected 1 of 4 slightly different versions of video in each session. Participants could also raise questions. The chatbot would forward these questions to project staff, who answered later through the messaging and video calling app or telephone (Figure 2).

### Study Outcomes

Self-reported PV uptake measured at T1 (12 months after completion of the intervention) was the primary outcome. This outcome was validated by requesting participants to upload an image of their PV receipt, concealing personal identification, via the messaging and video calling app number used in this study. No incentive was offered for validating PV uptake. The secondary outcome was participants’ stage of change measured at T0 (baseline) and T1 by using validated questions.^23^ The measurements and definitions of stage of change were the same as those used by the chatbot. Engagement with the chatbot was also measured. The number of intervention sessions received and completed by each participant and number of questions asked by the participants in the stage of change group were retrieved from the chatbot system. The participants’ subjective experience with the chatbot was measured by the validated TWEETS (TWente Engagement with Ehealth Technologies Scale) at T1.^24^

### Statistical Analysis

There were no missing data for participants who completed the surveys at T0 and T1. The proportion of missing data at T1 was equal to the dropout rate. An intention-to-treat analysis was performed. All randomized participants were included in the analysis even if they did not receive the assigned intervention or dropped out from the study. Participants were analyzed according to the group they were originally assigned to. Multiple imputation (5 times) was used to replace the missing outcomes at T1. Assuming the data were missing at random, the Markov Chain Monte Carlo method was used to impute missing primary and secondary outcomes at T1 separately by each group.^25^ Variables used to impute missing values included background characteristics that strongly predicted PV uptake in the literature (sex, age, and seasonal influenza vaccination uptake) and baseline values of such outcomes.^6^ The relative risk, absolute risk reduction, and number needed to treat and their respective 95% CIs were calculated using Excel without adjusting for covariates. Between-group differences in baseline characteristics were compared using χ^2^ tests or independent-samples *t* tests. In the stage of change group, the difference between the stage of change documented by the chatbot at the last intervention session vs the first intervention session was assessed using a paired-samples *t* test. The significance level was set as a 2-sided *P* < .05. Analyses were conducted using SPSS, version 26.0 (SPSS Inc), and R, version 4.2.2 (R Project for Statistical Computing).

## Results

**Figure 3.**
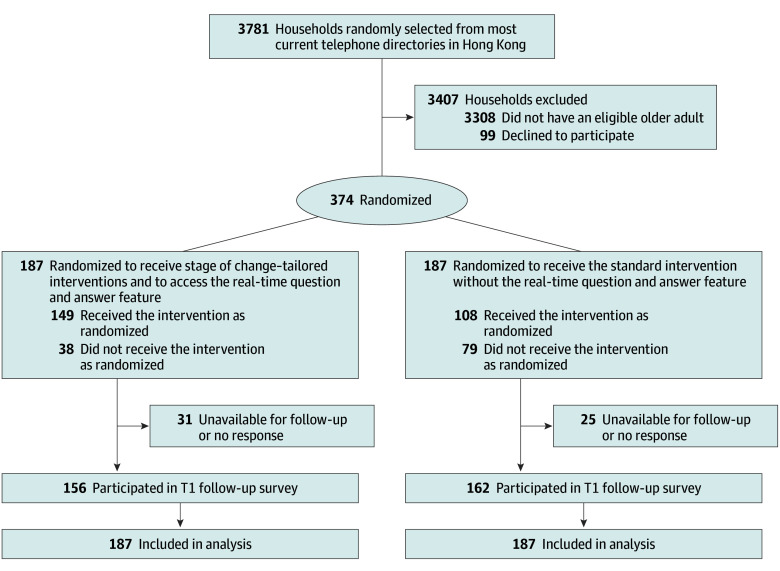
CONSORT Flow Diagram of Participant Recruitment and Retention T1 indicates 12 months after completion of the intervention.

From May 1 to September 16, 2023, 3781 households were contacted to recruit RCT participants (Figure 3). Of them, 482 had an eligible older adult, and 374 individuals completed the baseline survey and were randomly assigned to the stage of change (n = 187) or standard intervention (n = 187) groups; 213 were female (57.0%) and 161 were male (43.0%), with a mean (SD) age of 69.6 (3.1) years (Table 1). The dropout rate at T1 (12 months after completion of the intervention) was 16.6% and 13.4% in the stage of change and standard intervention groups, respectively. Comparison of the baseline characteristics between dropouts and nondropouts is provided in eTable 1 in Supplement 1.

**Table 1.** Characteristics of Participants at Baseline

Characteristic	Participants, No. (%)		
	All (n = 374)	Stage of change (n = 187)	Standard intervention (n = 187)
Age group, y			
65-69	260 (69.5)	135 (72.2)	125 (66.8)
70-74	81 (21.7)	36 (19.3)	45 (24.1)
≥75	33 (8.8)	16 (8.6)	17 (9.1)
Sex assigned at birth			
Female	213 (57.0)	103 (55.1)	110 (58.8)
Male	161 (43.0)	84 (44.9)	77 (41.2)
Relationship status			
Single	98 (26.2)	46 (24.6)	52 (27.8)
Married or cohabiting with a partner	276 (73.8)	141 (75.4)	135 (72.2)
Educational level			
Primary or below	80 (21.4)	40 (21.4)	40 (21.4)
Secondary	201 (53.7)	100 (53.5)	101 (54.0)
Tertiary or above	93 (24.9)	47 (25.1)	46 (24.6)
Full- or part-time employment			
No	293 (78.3)	146 (78.1)	147 (78.6)
Yes	81 (21.7)	41 (21.9)	40 (21.4)
Monthly household income, HK$ (US$)			
<20 000 (2580)	168 (44.9)	83 (44.4)	85 (45.5)
≥20 000 (2580)	113 (30.2)	62 (33.2)	51 (27.3)
Declined to disclose	93 (24.9)	42 (22.5)	51 (27.3)
Receiving CSSA^a^			
No	360 (96.3)	183 (97.9)	177 (94.7)
Yes	14 (3.7)	4 (2.1)	10 (5.3)
Living alone			
No	324 (86.6)	167 (89.3)	157 (84.0)
Yes	50 (13.4)	20 (10.7)	30 (16.0)
Smoking in the past year			
No	361 (96.5)	181 (96.8)	180 (96.3)
Yes	13 (3.5)	6 (3.2)	7 (3.7)
Binge alcohol consumption in the past year			
No	366 (97.9)	182 (97.3)	184 (98.4)
Yes	8 (2.1)	5 (2.7)	3 (1.6)
Presence of a chronic condition, yes			
Hypertension	151 (40.4)	83 (44.4)	68 (36.4)
Chronic cardiovascular disease	18 (4.8)	9 (4.8)	9 (4.8)
Chronic lung disease	2 (0.5)	0	2 (1.1)
Chronic liver disease	7 (1.9)	2 (1.1)	5 (2.7)
Chronic kidney disease	0	0	0
Diabetes	51 (13.6)	25 (13.4)	26 (13.9)
History of confirmed SARS-CoV-2 infection			
No	114 (30.5)	59 (31.6)	55 (29.4)
Yes	260 (69.5)	128 (68.4)	132 (70.6)
Received seasonal influenza vaccination in 2022-2023 flu season (October 2022 to September 2023)			
No	225 (60.2)	117 (62.6)	108 (57.8)
Yes	149 (39.8)	70 (37.4)	79 (42.2)
No. of doses of COVID-19 vaccination			
≥3	329 (88.0)	161 (86.1)	168 (89.8)
2	30 (8.0)	16 (8.6)	14 (7.5)
0-1	15 (4.0)	10 (5.3)	5 (2.7)
Stage of change related to pneumococcal vaccination uptake			
Precontemplation	231 (61.8)	117 (62.6)	114 (61.0)
Contemplation	113 (30.2)	56 (29.9)	57 (30.5)
Preparation	30 (8.0)	14 (7.5)	16 (8.6)
Stage of change score, mean (SD)^b^	1.5 (0.6)	1.4 (0.6)	1.5 (0.7)

Abbreviation: CSSA, Comprehensive Social Security Assistance.

^a^ CSSA provides a safety net for Hong Kong residents who cannot support themselves financially to meet their basic needs.

^b^ Stage of change score: 1 = precontemplation, 2 = contemplation, 3 = preparation, and 4 = action.

At T1, 55 participants in the stage of change group and 35 in the standard intervention group received PV during the study period, and all of them provided receipts for verification. In the intention-to-treat analysis, the PV uptake rate in the stage of change group was significantly higher than that in the standard intervention group (29.4% vs 18.7%; relative risk, 1.57 [95% CI, 1.08-2.28]; absolute risk reduction, 0.11 [95% CI, 0.02-0.19]; and number needed to treat, 9.4 [95% CI, 5.2-47.7]; *P* = .01). The mean (SD) score for the stage of change was higher in the stage of change group than in the standard intervention group (2.2 [1.3] vs 1.9 [1.1]; *P* = .02) (Table 2). Among 93 participants in the stage of change group who had completed at least 2 intervention sessions, a significant increase in their mean (SD) stage of change score was observed (2.1 [1.2] vs 1.3 [0.6]; *P* < .001) (eFigure 2 in Supplement 1).

**Table 2.** Between-Group Differences in Pneumococcal Vaccination Uptake and Stage of Change Related to Pneumococcal Vaccination

Outcome	Participants, No. (%)		*P* value^a^
	Stage of change group	Standard intervention group	
No. of participants included in the analysis	187	187	
Pneumococcal vaccination uptake, yes	55 (29.4)	35 (18.7)	.01
Stage of change 12 mo after intervention completion			
Precontemplation	84 (44.9)	95 (50.8)	.048
Contemplation	30 (16.0)	46 (24.6)	
Preparation	17 (9.1)	11 (5.9)	
Action	55 (29.4)	35 (18.7)	
Stage of change score, mean (SD)^b^	2.2 (1.3)	1.9 (1.1)	.02

^a^ *P* values obtained from χ^2^ tests (for pneumococcal vaccination uptake and categorical variable of stage of change) and independent-samples *t* test (for continuous variable of stage of change).

^b^ Stage of change score: 1 = precontemplation, 2 = contemplation, 3 = preparation, and 4 = action.

As documented by the chatbot system, more participants in the stage of change group than in the standard intervention group completed at least 1 intervention episode (79.7% vs 57.8%; *P* < .001). The mean (SD) number of intervention sessions completed by the participants was 1.6 (1.2) in the stage of change group and 1.5 (1.6) in the standard intervention group. In the stage of change group, 70 participants raised 164 questions (range, 1-9 questions); 9 questions were outside the Q&A database. In the standard intervention group, 24 participants raised 24 questions. A project staff answered questions outside the Q&A database or questions raised by the standard intervention group within 0.5 to 24 hours. Subjective engagement with the chatbot is presented in eTable 2 in Supplement 1.

## Discussion

This RCT evaluated the efficacy of a hybrid chatbot as a new approach to improve PV uptake among older adults. The chatbot-delivered interventions addressed some main modifiable barriers to uptake of PV among older adults, including lack of awareness about benefits and low self-efficacy, and concerns about adverse effects, high cost, and inconvenience related to PV.^6^ In line with our hypothesis, PV uptake was higher in the stage of change group than in the standard intervention group 12 months after completion of the intervention. In a relevant study, a natural language processing chatbot was developed for caregivers in Pakistan to answer queries related to childhood vaccination, including PV.^26^ However, no significant difference in children’s PV uptake was observed between chatbot users and nonusers. Compared with existing effective interventions promoting PV among older adults,^7,8,9,10^ our chatbot was fully automated and required fewer resources to implement.

Several reasons may explain the higher PV uptake rate in the stage of change group compared with the standard intervention group. First, the stage of change–tailored interventions facilitated the progression of participants to a higher stage of change, which may have led to the observed behavioral changes.^27^ Second, interventions tailored to users’ stage of change are more likely to create personal relevance, an essential component for effective interpersonal health communication.^28^ Moreover, the hybrid chatbot provided real-time answers to questions raised by participants in the stage of change group, which could increase engagement with digital health interventions.^15^ We found that more participants in the stage of change group than in the standard intervention group completed at least 1 intervention episode. Better compliance with assigned chatbot-delivered interventions has been associated with higher motivation to receive vaccination.^29^ Therefore, the observed efficacy may reflect additive or synergetic effects of stage of change–tailored content and the real-time Q&A function.

Few vaccine chatbots have been evaluated in low- and middle-income-countries.^26,30^ Most vaccine chatbots are operated via smartphones, which presents challenges of accessibility. Moreover, these vaccine chatbots required some digital health literacy to operate, which could be challenging for individuals of low socioeconomic status. Future studies should explore the feasibility and effectiveness of vaccine chatbots in these countries.

### Limitations

This study has limitations. First, this study did not assess whether using real-time Q&A functions alone could increase PV uptake. Second, the intervention was limited to adults 65 years of age or older who had access to smartphones. However, given the high smartphone ownership (96.4%) and popularity of the messaging and video calling app, accessibility was not an issue for older adults in Hong Kong.^31^ Third, people 75 years of age or older were undersampled in this study.^32^ Fourth, 22.5% of eligible older adults declined to join the study; thus, we were unable to collect their information. Participants who took part in the study and individuals who declined to participate may have different motivations to receive PV, and self-selection bias existed. Fifth, the stage of change was self-reported and could not be validated. Participants may have overreported their stage of change due to social desirability. Health conditions and other vaccination histories were self-reported. We were unable to access participants’ medical records for validation. Recall bias existed. Furthermore, due to the difference in vaccination delivery models, digital literacy, and cultural factors, the findings may not be applicable to other settings.

## Conclusions

This RCT showed that a hybrid chatbot was more effective than a chatbot-delivered standard intervention in increasing PV uptake among community-dwelling individuals 65 years of age or older. A hybrid chatbot may be a sustainable approach for promoting PV among older adults.

## Notes

Supplement 1. **eAppendix 1.** Epidemiology of invasive pneumococcal diseases and schedule of pneumococcal vaccination in Hong Kong **eAppendix 2.** Description of stage of change model **eAppendix 3.** Development of the chatbot and online intervention **eFigure 1.** Architecture of the chatbot system **eTable 1.** Comparing baseline characteristics between participants that completed month 12 evaluation and those that were unavailable for follow-up **eFigure 2.** Stages of changes documented by the chatbot at the first and the last intervention session in the stage of change group **eTable 2.** Subjective experience of engagement with the chatbot among users in the stage of change group and the standard intervention group

Supplement 2.
Trial Protocol

Supplement 3.
Data Sharing Statement
