# Supplementary material for: A Hybrid Chatbot to Promote Pneumococcal Vaccination Among Older Adults: A Randomized Clinical Trial
Source: JAMA Netw Open. 2025 Oct 8;8(10):e2535813. doi: 10.1001/jamanetworkopen.2025.35813 (PMC12509012; doi:10.1001/jamanetworkopen.2025.35813)
Supplement: Supplement 1. — eAppendix 1. Epidemiology of invasive pneumococcal diseases and schedule of pneumococcal vaccination in Hong Kong eAppendix 2. Description of stage of change model eAppendix 3. Development of the chatbot and online intervention eFigure 1. Architecture of the chatbot system eTable 1. Comparing baseline characteristics between participants that completed month 12 evaluation and those that were unavailable for follow-up eFigure 2. Stages of changes documented by the chatbot at the first and the last intervention session in the stage of change group eTable 2. Subjective experience of engagement with the chatbot among users in the stage of change group and the standard intervention group [file jamanetwopen-e2535813-s001.pdf]

Supplementary Online Content

Wang Z, Chen S, Poon J, et al. A hybrid chatbot to promote pneumococcal vaccination among older adults: a randomized clinical trial. *JAMA Netw Open*. 2025;8(10):e2535813. doi:10.1001/jamanetworkopen.2025.35813

eAppendix 1. Epidemiology of invasive pneumococcal diseases and schedule of pneumococcal vaccination in Hong Kong

eAppendix 2. Description of stage of change model

eAppendix 3. Development of the chatbot and online intervention

eFigure 1. Architecture of the chatbot system

eTable 1. Comparing baseline characteristics between participants that completed month 12 evaluation and those that were unavailable for follow-up

eFigure 2. Stages of changes documented by the chatbot at the first and the last intervention session in the stage of change group

eTable 2. Subjective experience of engagement with the chatbot among users in the stage of change group and the standard intervention group

This supplementary material has been provided by the authors to give readers additional information about their work.

© 2025 Wang Z et al. *JAMA Network Open*.

eAppendix 1. Epidemiology of invasive pneumococcal diseases and schedule of pneumococcal vaccination in Hong Kong

(1) Epidemiology of invasive pneumococcal diseases in Hong Kong

In Hong Kong, the number of IPD cases was relatively stable before the time of COVID-19. During 2007 and 2019, 117 to 205 IPD cases per year were reported, with an annual incidence between 1.67 and 2.84 per 100,000 persons. During the COVID-19 pandemic and due to the control measures, the number of IPD cases dropped to 47, 25, and 28 in 2020, 2021, and 2022, with an annual incidence from 0.34 to 0.63 per 100,000 persons. After the COVID-19 control measures were lifted, the number of reported IPD cases increased in 2023 (67 cases) [1].

People aged 65 years or above in Hong Kong had higher risk of IPD. The incidence of IPD in this population was between 5 to 10 per 100,000 persons during 2007 to 2014, and slightly decreased from 6.73 per 100,000 persons in 2015 to 5.57 per 100,000 persons in 2019. Such incidence decreased to about 2 per 100,000 persons during the COVID-19 pandemic (2020-2022) and rebounded to approximately 4 per 100,000 persons in 2023. In terms of mortality of IPD, 124 death cases were reported in adults aged 65 years or above from 2015 to 2022, with a case fatality rate ranged from 21.4% to 40% [1].

Subtypes covered by PCV13 accounted for 48-75% of IPD cases each year during 2015-2023. Serotype 3 is the most common serotype of IPD, followed by 19A and 14. All these three serotypes are covered by PCV13. Serotypes 22F (17 cases, accounted for 1.6% of all reported cases between 2015 and 2023), which is covered by PCV15 but not by PCV13, was also reported in Hong Kong. Seven serotypes (8, 10A, 11A, 12F, 15B, 22F, and 33F) are unique to PCV20 in addition to those covered by PCV13, contributed to 6.4% of IPD cases reported during 2015-2023. In addition, non-vaccine serotypes accounted for 17.2% of all IPD cases during the same report period [1].

(2) Pneumococcal vaccination schedule in Hong Kong

people aged 65 years or above without high risk conditions listed by the Centre for Protection (history of pneumococcal diseases, cerebrospinal fluid leakage, cochlear implant, chronic cardiovascular, lung, liver or kidney diseases, metabolic diseases or obesity,

© 2025 Wang Z et al. *JAMA Network Open*.

immunocompromised states and chronic neurological conditions that can compromise respiratory functions) are recommended to receive one dose of 23-valent pneumococcal conjugate vaccine (23vPPV). Persons with the aforementioned high-risk conditions are recommended to receive one dose of 13-valent pneumococcal conjugate vaccine (PCV13), followed by one dose of 23vPPV one year after. Starting from 5 August 2024, PCV13 has been replaced by PCV15, while other recommendation remained unchanged [2].

Reference

[1] Centre for Health Protection. Communicable Diseases Watch. Available at: https://www.chp.gov.hk/files/pdf/cdw_v19_8.pdf. Accessed on July 16, 2025

[2] Centre for Health Protection. Pneumococcal vaccination. Available at: https://www.chp.gov.hk/en/features/108124.html. Accessed on July 16, 2025

© 2025 Wang Z et al. *JAMA Network Open*.

eAppendix 2. Description of stage of change model

The stage of change model is one of the most commonly used stage models, which postulates that completed behavioural change will go through five ordinal stages: precontemplation, contemplation, preparation, action and maintenance [1].

In the context of vaccination behaviors, individuals who are in the contemplation stage do not consider taking up the vaccines [2]. Those in the contemplation stage would consider receiving the vaccines but have not committed to take action [2]. People at the preparation stage have plans to receive the vaccines in near future and may have taken some initial steps (e.g., making appointment) [2]. The action stage refers to individuals who have already received the vaccines [2].

The stage of change model suggests that intervention should be tailored to an individual's current stage to promote sustained behavioral change [1]. For example, individuals in the pre-contemplation stage may need interventions focused on increasing their awareness of potential behavioural changes by providing information and explaining reasons for making changes. For those who are at the contemplation stage, strategies include elicit benefits and costs to shift their decisional balance in favour or benefits, make specific suggestion, and encourage to make specific plan for taking action. A meta-analysis supported that interventions tailored to one's current stage of change were more effective in promoting vaccination uptake than non-stage of change-tailored interventions or no interventions [3].

References
[1] Prochaska JO & Velicer WF. The transtheoretical model of health behavior change. American Journal of Health Promotion, 1997; 12(1): 38-48
[2] Norcross JC, et al. Stage of change. Journal of Clinical Psychology, 2011; 67(2): 143-154
[3] Chen S, et al. Effectiveness of stage of change (SOC)-tailored interventions in increasing uptake of any type of vaccination: a systematic review and meta-analysis. Applied Psychology – Health and Wellbeing. 2025, 17(2): e70022

© 2025 Wang Z et al. *JAMA Network Open*.

eAppendix 3. Development of the chatbot and online intervention

(1) Co-design the intervention materials and the chatbot system

We used a co-design approach to develop the intervention materials and the chatbot system. The co-design approach is based on a 3-stage model developed by the National Health Service of United Kingdom government [1].

The first stage was to define needs and experiences of the users and stakeholder. The research team conducted comprehensive literature search and in-depth interviews of five local older adults to identify facilitators and barriers to receiving the PV among older adults in Hong Kong. The in-depth interviews also explored older adults' preferences about the format of interventions, concerns, and frequently asked questions (FAQ) related to pneumococcal vaccination (PV). Fieldnotes were taken immediately after each interview. The interviews were transcribed for thematic analysis. Key suggestions made by older adults included using both videos and texts to deliver health communication messages, and allowing flexible input options to interact with the chatbot.

The second stage was to develop ideas and prototype. A panel involving both investigators (experts in public health and vaccination behaviours, physicians, and health psychologists) and five older adults held multiple meetings, taking the findings in the literature review, our groundwork and in-depth interviews for creating health communication messages based on the behavioural change strategies recommended by the SOC to address the known determinants of PV uptake, preparing the chatbot workflow, and comprehensive question-answer (QA) pairs.

The final stage was to conduct iterative testing and delivery. Discussion groups involving another five older adults were conducted to enrich intervention materials concerning relevance and usefulness of the health communication messages, and to review the chatbot workflow and QA pairs. Their input was discussed in panel meetings to finalize the workflow and the key QA database. Another 10 older adults were invited to test the chatbot. With informed consent, the research team retrieved and reviewed users' chat history with the chatbot. The chatbot was running smoothly during pilot testing, and all users were satisfied with its performance.

(2) Architecture of the chatbot

Our chatbot is an NLP-based chatbot and is not publicly available, only participants of this study had access to the chatbot during the project period. We integrated the chatbot with the

WhatsApp platform via its public Web API (Figure 1). Participants' messages are routed through WhatsApp's instant messaging server to a separately constructed chatbot system, comprising both an administrative interface and the chatbot itself. Upon processing, the chatbot's response is returned to the WhatsApp server and immediately visible to the user. This process is virtually instantaneous, delivering a seamless user experience. The chatbot system comprises three primary modules: i) Dialogue management module: This module logs all interactions between users and the chatbot, capturing essential details about users' activity and previous exchanges. The system's natural language processing component interprets each message's content, forwarding it to trigger relevant actions based on predefined rules. For example, if a message includes a specific keyword, the chatbot automatically replies with the corresponding information. Additionally, the module can initiate new conversations according to the intervention schedule; ii) User management module: This module tracks user engagement by recording conversation details. Administrators can associate users' WhatsApp numbers with the chatbot, monitor intervention progress, and identify any disconnections; and iii) Multimedia management module: Supporting media functionality, this module enables the chatbot to handle image and video exchanges, allowing users to upload, send, and receive multimedia content as part of their interactions.

(3) Conversation mechanism of the hybrid chatbot

Our chatbot used pre-defined rules to deliver co-designed stage of change (SOC)-tailored interventions. The chatbot uses NLP functions to interpret and understand users' input/questions, and retrieves most relevant responses from the key QA database using keyword matching. If the chatbot cannot match the user's input with a relevant response, it replies, "I am not confident about an answer to your question." After three consecutive off-topic inputs, the chatbot suggests a new conversation topic. When users request additional information on a topic, the chatbot provides varied responses within the key QA database to add depth to the topic, and it can repeat responses upon request. If a conversation is interrupted, the chatbot cancels any incomplete inputs and invites the user to re-enter their message to ensure clarity and continuity. The chatbot was trained to answer questions based on the key QA database as accurately as possible until convergence was reached.

(4) Data safety

Participants' chat history was protected by the confidential agreement with WhatsApp. The data was encrypted on the chatbot server, and would not be used other parties. The chat history

© 2025 Wang Z et al. *JAMA Network Open*.

was removed from the server once the project is completed. Until then, the participants' chat history was stored in the chatbot server and protected by passwords, with only the principal investigator having access to the data. With these measures, we believed the risk of a data breach should be minimal.

References

[1]. Kilfoy, A., Hsu, TC.C., Stockton-Powdrell, C. *et al.* An umbrella review on how digital health intervention co-design is conducted and described. *npj Digit. Med.* **7**, 374 (2024). https://doi.org/10.1038/s41746-024-01385-1".

© 2025 Wang Z et al. *JAMA Network Open*.

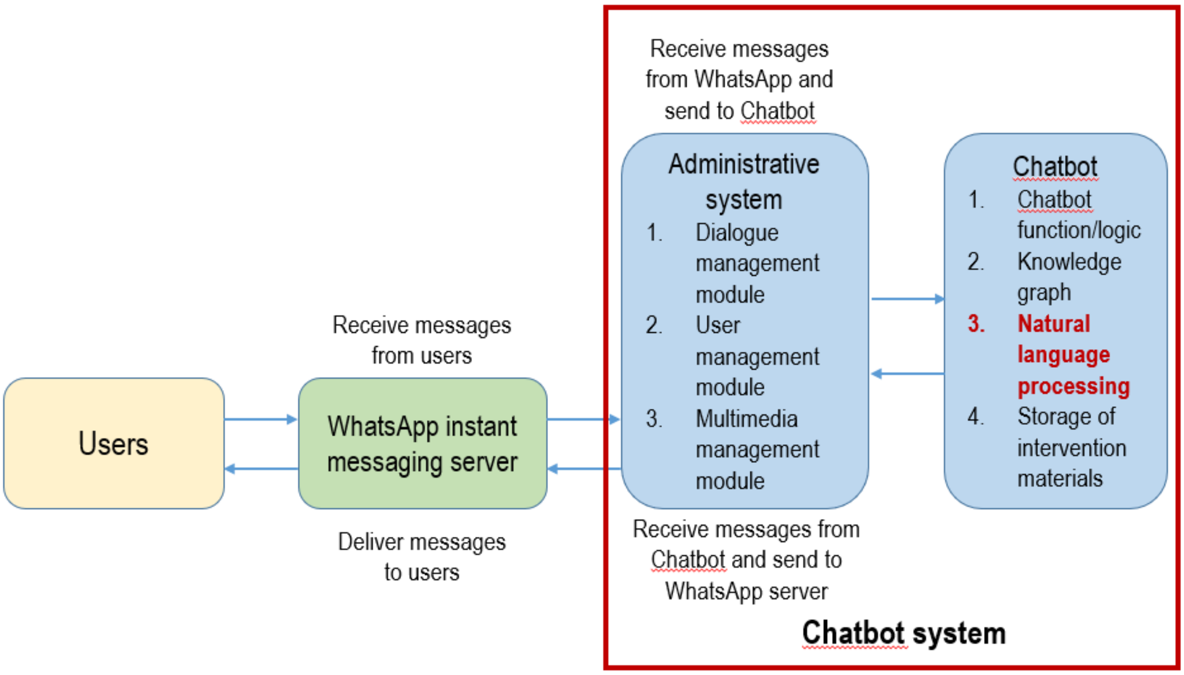

eFigure 1. Architecture of the chatbot system

© 2025 Wang Z et al. *JAMA Network Open*.

eTable 1. Comparing baseline characteristics between participants that completed month 12 evaluation and those that were unavailable for follow-up

	Stage of change group (n=187)			Standard intervention group (n=187)		
	Being followed-up (n=156)	Unavailable for follow-up (n=31)	P values	Being followed-up (n=162)	Unavailable for follow-up (n=25)	P values
	n (%)	n (%)		n (%)	n (%)	
**Socio-demographics**						
Age group, years			0.19			0.11
65-69	113 (72.4)	22 (71.0)		104 (64.2)	21 (84.0)	
70-74	32 (20.5)	4 (12.9)		43 (26.5)	2 (8.0)	
≥75	11 (7.1)	5 (16.1)		15 (9.3)	2 (8.0)	
Sex assigned at birth			0.67			0.90
Male	69 (44.2)	15 (48.4)		67 (41.4)	10 (40.0)	
Female	87 (55.8)	16 (51.6)		95 (58.6)	15 (60.0)	
Relationship status			0.23			0.35
Single	41 (26.3)	5 (16.1)		47 (29.0)	5 (20.0)	
Married or cohabiting with a partner	115 (73.7)	26 (83.9)		115 (71.0)	20 (80.0)	
Education level			0.16			0.84
Primary or below	35 (22.4)	5 (16.1)		34 (21.0)	6 (24.0)	
Secondary	86 (55.2)	14 (45.2)		87 (53.7)	14 (56.0)	
Tertiary or above	35 (22.4)	12 (38.7)		41 (25.3)	5 (20.0)	
Monthly household income, HK$ (US$)			0.89			0.24
<20,000 (2,564)	70 (44.9)	13 (41.9)		77 (47.5)	8 (32.0)	
≥20,000 (2,564)	52 (33.3)	10 (32.3)		44 (27.2)	7 (28.0)	
Refuse to disclose	34 (21.8)	8 (25.8)		41 (25.3)	10 (40.0)	
Receiving CSSA [1]			0.65			0.20
No	153 (98.1)	30 (96.8)		152 (93.8)	25 (100)	
Yes	3 (1.9)	1 (3.2)		10 (6.2)	0 (0.0)	
Living alone			0.40			1.00
No	138 (88.5)	29 (93.5)		136 (84.0)	21 (84.0)	
Yes	18 (11.5)	2 (6.5)		26 (16.0)	4 (16.0)	
**Lifestyles and health conditions**						
Smoking in the past year			1.00			0.29
No	151 (96.8)	30 (96.8)		155 (95.7)	25 (100)	
Yes	5 (3.2)	1 (3.2)		7 (4.3)	0 (0.0)	
Binge drinking in the past year			0.15			0.49
No	153 (98.1)	29 (93.5)		159 (98.1)	25 (100)	
Yes	3 (1.9)	2 (6.5)		3 (1.9)	0 (0.0)	
Presence of some chronic condition, yes						
Hypertension	70 (44.9)	13 (41.9)	0.76	58 (35.8)	10 (40.0)	0.69
Chronic cardiovascular diseases	8 (5.1)	1 (3.2)	0.65	9 (5.6)	0 (0.0)	0.23
Chronic lung diseases	0 (0.0)	0 (0.0)	N.A.	2 (1.1)	0 (0.0)	0.58
Chronic liver diseases	1 (0.6)	1 (3.2)	0.20	5 (3.1)	0 (0.0)	0.37
Chronic kidney diseases	0 (0.0)	0 (0.0)	N.A.	0 (0.0)	0 (0.0)	N.A.
Diabetes	21 (13.5)	4 (12.9)	0.93	22 (13.6)	4 (16.0)	0.75
History of confirmed SARS-CoV-2 infection			0.93			0.44
No	49 (31.4)	10 (32.3)		46 (28.4)	9 (36.0)	
Yes	107 (68.6)	21 (67.7)		116 (71.6)	16 (64.0)	
**Vaccination history**						

© 2025 Wang Z et al. *JAMA Network Open*.

Receiving seasonal influenza vaccination in the 2022/2023 flu season (October 2022 – September 2023)			0.87			0.81
No	98 (62.8)	19 (61.3)		93 (57.4)	15 (60.0)	
Yes	58 (37.2)	12 (38.7)		69 (42.6)	10 (40.0)	
Number of doses of COVID-19 vaccination			0.60			0.46
≥3	136 (87.2)	25 (80.6)		146 (90.1)	22 (88.0)	
2	12 (7.7)	4 (12.9)		11 (6.8)	3 (12.0)	
0-1	8 (5.1)	2 (6.5)		5 (3.1)	0 (0.0)	
**Stages-of-change**						
Stage of change related to pneumococcal vaccination uptake			0.61			0.68
Precontemplation stage	97 (62.2)	20 (64.5)		98 (60.5)	16 (64.0)	
Contemplation stage	46 (29.5)	10 (32.3)		49 (30.2)	8 (32.0)	
Preparation stage	13 (8.3)	1 (3.2)		15 (9.3)	1 (4.0)	
SOC score, mean (SD) [2]	1.5 (0.6)	1.4 (0.6)	0.55	1.5 (0.7)	1.4 (0.6)	0.53

[1] CSSA: Comprehensive Social Security Assistance Scheme, which provides a safety net for Hong Kong residents who cannot support themselves financially to meet their basic needs

[2] Stage of change score: 1= precontemplation stage, 2=contemplation stage, 3=preparation stage, & 4=action stage

© 2025 Wang Z et al. *JAMA Network Open*.

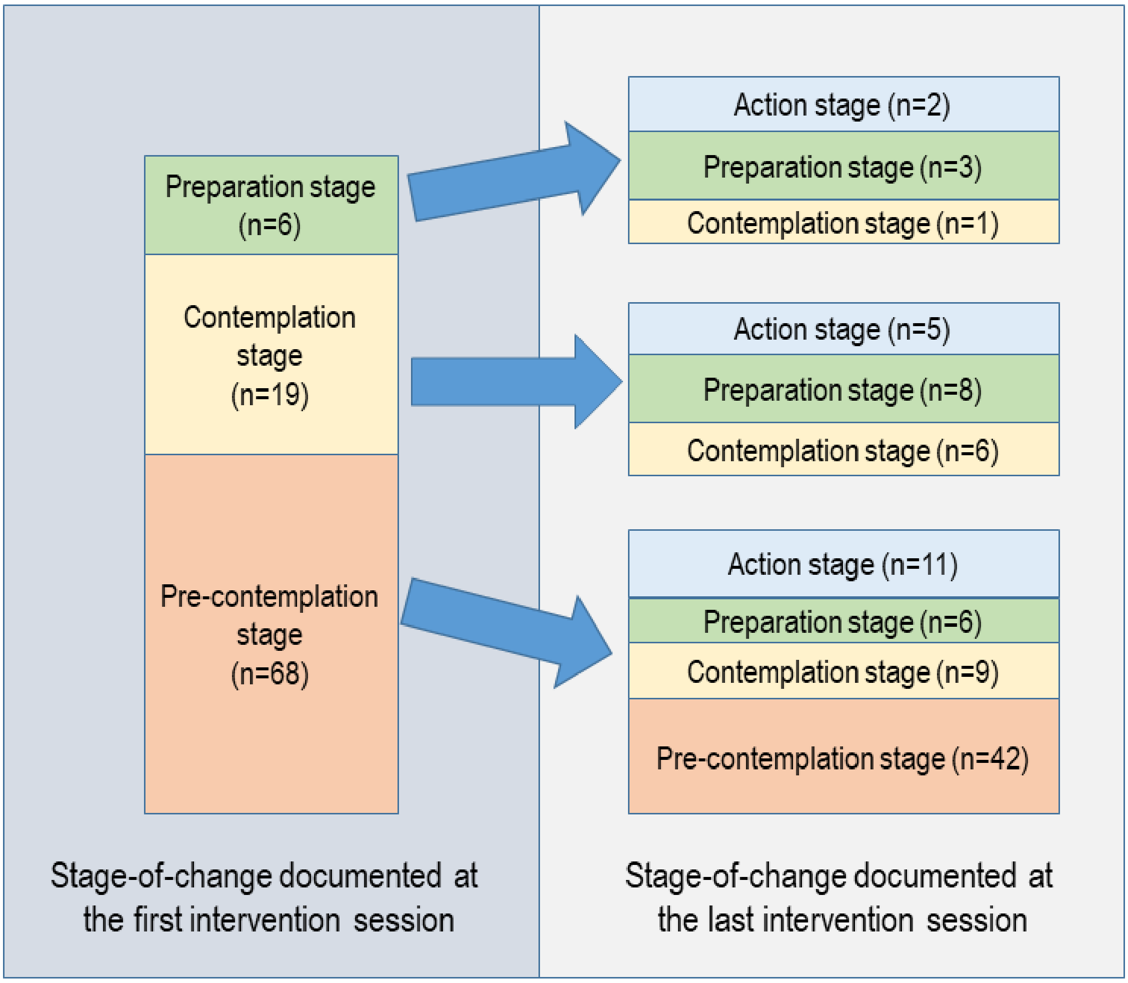

eFigure 2. Stages of changes documented by the chatbot at the first and the last intervention session in the stage of change group

© 2025 Wang Z et al. *JAMA Network Open*.

eTable 2. Subjective experience of engagement with the chatbot among users in the stage of change group and the standard intervention group

	Chatbot users in the stage of change group (n=143)	Chatbot users in the standard intervention group (n=99)	*P* values
	n (%)	n (%)	
Behavioural engagement, strongly agree/agree			
The chatbot is easy to use	117 (81.8)	74 (74.7)	.19
You are able to use the chatbot as often as you needed	120 (83.9)	72 (72.7)	.04
Cognitive engagement, strongly agree/agree			
The chatbot makes it easier for me to work on my goal	81 (56.6)	59 (59.6)	.65
The chatbot motivates me to receive pneumococcal vaccination	88 (61.5)	56 (56.6)	.44
The chatbot helps me to get more insight into pneumococcal vaccination	112 (78.3)	71 (71.7)	.24
Affective engagement, strongly agree/agree			
You enjoyed using the chatbot	120 (83.9)	78 (78.8)	.31
I enjoyed seeing the progress I made when using the chatbot	102 (71.3)	68 (68.7)	.66
The chatbot fits me as a person	119 (83.2)	79 (79.8)	.50

P values were obtained using Chi-square tests

© 2025 Wang Z et al. *JAMA Network Open*.
