# Supplementary material for: A Hybrid Chatbot to Promote Pneumococcal Vaccination Among Older Adults: A Randomized Clinical Trial
Source: JAMA Netw Open. 2025 Oct 8;8(10):e2535813. doi: 10.1001/jamanetworkopen.2025.35813 (PMC12509012; doi:10.1001/jamanetworkopen.2025.35813)
Supplement: Supplement 2. — Trial Protocol [file jamanetwopen-e2535813-s002.pdf]

13. PROPOSED RESEARCH PROJECT

This template applies to the following Area of Project* –

- **Public health, human health and health services research**
- **Infectious diseases**
- **Advanced medical research**

**** For health promotion project, please use another template.***

a) Title:
A randomized controlled trial evaluating a theory-based online intervention via fully automated Chatbot in increasing pneumococcal vaccination among community dwelling individuals aged ≥65 years

b) Introduction:

Pneumococcal diseases (PD) --- a serious public health threat

Streptococcus pneumoniae causes a wide spectrum of diseases and is the most common cause of community-acquired pneumonia (1). The infection can also spread into the blood stream causing invasive PD (IPD) (1). In Hong Kong, pneumonia is the second leading cause of death in 2018 (n=8,334) (2). Individuals aged ≥65 years are at much higher risk of PD and IPD and had the highest risk of death from IPD (3). In Hong Kong, 39.6% (75/189) of IPD were reported in this age group (4). Elderly with the following health conditions listed by the Hong Kong Centre for Health Protection (CHP) are more vulnerable to severe IPD: 1) history of IPD, 2) immunocompromised states, 3) chronic diseases (chronic cardiovascular, lung, liver or kidney diseases, diabetes mellitus, and cerebrospinal fluid leakage), and 4) cochlear implant (5). Moreover, secondary and co-infection of bacterial pneumonia (mostly caused by *Streptococcus pneumoniae*) following seasonal influenza is common among elderly and associated with higher mortality and morbidity (6).

Pneumococcal vaccination (PV) for elderly --- an important health initiate

The 13-valent pneumococcal conjugate vaccines (PCV13) and the 23-valent pneumococcal polysaccharide vaccine (23vPPV) are effective in preventing vaccine-type community-acquired pneumonia (44.8-45.6% protection), pneumococcal pneumonia (63.8% protection) and IPD (65-75% protection) among individuals aged ≥65 years (7). Receiving PV in addition to seasonal influenza vaccination can prevent additional hospitalization and deaths in elderly (8). Both types of PV are available in Hong Kong and their safety is well documented (7).

International and local PV programs for elderly

Health authorities in Hong Kong and many other jurisdictions recommend PV for individuals aged ≥65 years (9). Hong Kong launched the Vaccination Subsidy Scheme and Government Vaccination Program in 2017. All community-living Hong Kong residents aged ≥65 years are eligible to receive free PV at public clinics and subsidized PV (PCV13: HK$730/dose; 23vPPV: HK$190/dose) at enrolled private clinics (5).

Groundwork performed by us

We conducted a random telephone survey of Hong Kong community-living individuals aged ≥65 years from May to July 2019 (10). Among the participants, 17.8% reported PV uptake, and 18.6% of unvaccinated participants intended to receive free PV in the next year. There is an urgent need and much room for improvement.

We found some perceptions that were associated with PV uptake. Facilitators included perceived high risk of PD/IPD, belief that PV could protect themselves and their family members, being suggested by significant others to receive PV, self-efficacy to receive PV, and having a peer of similar age who received PV. Barriers were concerns about cost, potential PV side effects, and inconvenience related to operating hours and location. The proposed intervention will modify these perceptions. This study will confirm the content by message testing. Associated factors of PV uptake identified by our study were similar to those found in literatures.

Interventions promoting PV among community-living elderly

To the best of our knowledge, only four community-based RCTs were conducted to promote PV among community-living elderly, although several others were targeting patients in clinic settings (see Appendix 1). These RCTs yielded mixed results in increasing PV uptake. One study compared six different interventions (short phone calls, SMS or email with and without patient education delivered by nurses via phone), but did not find significant between-group difference in PV uptake. Three other RCTs compared efficacies of telephone education session delivered by volunteer or nurses, or home visit paid by nurses versus the control condition (usual care). Significantly higher PV uptake was found in the intervention group of these three RCTs.

For interventions promoting PV to be effective and sustainable, it is important that such programs would not require extensive manpower. However, all aforementioned interventions required large number of phone/face-to-face education sessions by nurses/volunteers, making them less sustainable. It is warranted to develop sustainable PV promotion that requires minimum manpower to maintain. This study has considered such requirement.

Stage-tailored and theory-based intervention promoting PV among elderly

The Trans-Theoretical Model (TTM) has been widely used to guide health promotion (11). It conceptualizes the process of behavioural change. Stage of change (SOC), the core of TTM, is a measure of readiness for behavioural change (11). SOC postulates that behavioural changes need to go through five ordinal stages: 1) pre-contemplation stage (do not intend to take action in the foreseeable future), 2) contemplation stage (intend to change in the foreseeable future), 3) preparation stage (intend to take action in the immediate future), 4) action stage, and 5) maintenance stage.

Meta-analysis showed that interventions tailored to one's current SOC are more effective than non-stage-tailored ones, especially among less-motivated individuals (12). Stage-tailored interventions

based on the SOC were successfully used to promote screening behaviours, smoking cessation, physical activities, dietary change, condom use and substance use, some of these interventions were conducted among elderly (13). **To our knowledge, only one study applied stage-tailored interventions to promote vaccination (HPV vaccination)** (14).

In addition, studies showed people might move forward the later SOC, go backward earlier SOC, or stay in the same SOC after exposing to health promotion. Therefore, it is highly recommended that stage-tailored interventions should have multiple sessions and each session tailors people's current SOC (15). Very few stage-tailored interventions based on the SOC had more than one session.

Strategies facilitating transition of SOC

Since PV uptake is one-off, we focus on pre-contemplation, contemplation and preparation stage. According to the theory, different strategies are recommended for people in different SOC (11):

1) **To facilitate transition from pre-contemplation stage to contemplation stage,** common strategies include increase their awareness of potential behavioural changes by providing information and explaining reasons for making changes.
2) **To facilitate transition from contemplation stage to preparation stage,** strategies include elicit pros and cons to shift their decisional balance in favour or pros, make specific suggestion, and encourage to make specific plan for taking action.
3) **To facilitate transition from preparation stage to action stage,** strategies include assist them develop and implement concrete action plan in order to increase their self-efficacy.

We apply these strategies to modify perceptions influencing PV uptake identified by our groundwork.

Using Chatbot with natural language processing (NLP) functions to deliver stage-tailored interventions promoting PV for elderly

A Chatbot is a computer software which interprets users' responses in natural spoken language and/or written forms, and then automatically selects and provides human-like dialogue according to the recent utterance (16). With the advance in artificial intelligence (AI), Chatbots can learn from previous human-machine interactions in order to increase the accuracy and quality of future interactions (17). Chatbots have good potential in delivering interventions to change health behaviours. A review suggested that Chatbots were feasible and effective in promoting physical activities, healthy lifestyles, mental health and medication adherence (18). A number of successful AI-Chatbots have entered the market (see Appendix 3). Chatbots demonstrate some advantages in behavioural intervention. They can reach and handle large number of users with minimum manpower, and are able to provide interventions tailored to users' conditions.

Our team developed a Chatbot without NLP functions and applied it in smoking cessation (19). Through WeChat, the Chatbot assessed users' current smoking status, and disseminated interventions (text messages, images, or videos) tailored to their smoking status. For example, the Chatbot asked participants to click a box indicating number of cigarette consumed in the past week. For those clicked

zero consumption, the Chatbot automatically sent health promotion messages strengthening maintenance of quitting. While for those with cigarette smoking, the Chatbot sent messages to motivate them to quit. The details was described by a recently published paper (19). After exposure to the Chatbot-delivered interventions, the self-reported and biochemically validated quit rate was 29.0% and 18.6% at Month 6. The average number of cigarettes consumed by the non-quitters decreased significantly from 9.1 per day at baseline to 6.1 per day at Month 6. Such self-reported and validated quit rate was higher than other smoking cessation interventions in Hong Kong (self-reported: 7.4-23.0% and validated: 3.2-11.4%) (see Appendix 4). Our ongoing project modified the Chatbot (without NLP functions) to promote seasonal influenza vaccination among Hong Kong elderly (HMRF19181152).

In collaboration with IT engineers (Co-I: Dr. Josiah Poon and Dr. Caren Han), we will develop a fully-automated Chatbot that can interpret natural spoken language (i.e., Cantonese). Dr. Poon successfully developed a Chatbot with NLP function to support course selection for the University of Sydney (see Appendix 4). **Chatbots with NLP functions are especially attractive to elderly, as they are more opt to use their voice than typing texts** (20). The Chatbot will assess participants' SOC regarding PV uptake and deliver tailored interventions in an interactive way. We will build a knowledge graph (a system linking questions and responses) with increasing amount of prior conversations. Depending upon the conversation history and current dialogue, the Chabot retrieves the suitable response and chooses an appropriate persuasion style to communicate with the user. The Chatbot can also learn from the big pool of prior conversation pattern through machine learning to improve its performance. This is a novel approach.

Online intervention and Chatbot are feasible and effective for health promotion among elderly

In literature, online interventions are feasible and effective in promoting physical activities, mental health, and chronic diseases self-care among elderly [e.g., (21)]. With simple training, elderly can successful navigate through online interventions (21). For example, after receiving instructions on how to use WhatsApp and Health Apps, Hong Kong elderly with cognitive frailty had minimum difficulties to engage in an online intervention, which comprised of goals setting, logging physical activity data, and receiving real-time feedbacks (22). Difficulties in using Chatbot-delivered intervention were also minimal and easily remedied among elderly with low socio-economic status in the U.S. (23). Moreover, systematic review showed that elderly reported good compliance with the required dosage of online interventions (21). Making use of Chatbot to deliver online interventions further increased the compliance (23), as Chatbot can proactively interactive with the participants.

Significance

Very few community-based interventions promoting PV among community-living elderly. The existing interventions were either less effective or too resource demanding to sustain. None of them was based on behavioural health theories. This study is novel for applying a Chatbot with NLP functions to deliver intervention tailored to participants' SOC, which will increase its efficacy. Such approach can reach out to general public at low cost and is potential sustainable.

c) Aims and Hypotheses to be Tested:

The proposed RCT will compare the efficacy of a Chatbot-delivered stage-tailored online intervention (intervention group) versus a Chatbot-delivered non-stage-tailored online intervention (control group) increasing uptake of PV 12 months after completion of the interventions among unvaccinated individuals aged ≥65 years. In the intervention group, the Chatbot will assess participants' SOC regarding PV uptake and deliver interventions tailor to their SOC through WhatsApp every month for four times. In the control group, the Chatbot will not assess participants' SOC. It provides a standard intervention (covering key information of PV) which is not tailoring to participants' SOC every month for four times. Participants will complete two telephone surveys at baseline (T0) and 12 months after completion of the interventions (T1).

Secondary objectives are to evaluate the relative efficacy of the intervention in increasing the following responses compared to the control group:

1) SOC related to PV uptake at T1, and

2) Engagement with the Chatbot. The number of intervention sessions received and completed by all participants and number of questions asked by the participants in the intervention group will be retrieved from the Chatbot system. The participants' subjective experience related to behavioural, cognitive and affective engagement with the Chatbot will also be measured.

We hypothesize that PV uptake rate will be higher in the intervention group than that of the control group at T1.

d) Plan of Investigation:

(i) Subjects Inclusion criteria are: 1) aged ≥65 years, 2) having Hong Kong ID, 3) able to speak and comprehend Cantonese, 4) have not received any PV, 5) having a smartphone, 6) having WhatsApp installed on their smartphones, and 7) able to send and read text/voice messages via smartphone. The interviewers will ask the participants to reply a simple question (e.g., what date is today?) via SMS/WhatsApp in text/voice message to verify the last inclusion criterion.

Exclusion criteria include: 1) blindness or deafness, 2) with known contradictions of PV (allergy to any diphtheria toxoid-containing vaccine), 3) having been diagnosed with major psychiatric illness (schizophrenia and bipolar disorder) or dementia, and 4) score ≤16 in the validated telephone version of the Cantonese Mini-mental State Examination (T-CMMSE). The T-CMMSE has been validated among Hong Kong older adults, which showed satisfactory reliability and validity. With the training providing by our Co-I (Prof. Phoenix Mo) who is a chartered psychologist, the interviewers will use this tool to screen participants' mental capacity through telephone. A cut-off score of ≤16 was suggested to discriminate between those with and without dementia. Similar approach to ensure older adults' mental capacity to consent to join the study.

(ii) Methods **Recruitment procedures**

Participants will be recruited through random telephone sampling; **the method has been used for recruitment local elderly in our intervention studies** (HMRF 15161231 & 19181152).

Telephone numbers will be selected from up-to-date Hong Kong telephone directories. Trained interviewers will conduct the telephone calls. If there is more than one person in the household who is aged ≥65 years, the one whose last birthday is closest to the interview date will be invited to join the study. This is to avoid contamination and introduction of extra confounding factors. Eligibility will be screened.

Prospective eligible participants are briefed about the study. Guarantees will be made on anonymity, right to quit at any time and that refusal will not affect their chance in using services. Participants will be asked: 1) whether they understand the briefing and 2) whether they are willing to participate. They are reminded that there is a hotline for enquiry during office hour. Since there will not be face-to-face contact and the study is anonymous, the interviewers will sign a form pledging that the participants have been fully informed about the study. Similar procedures were used in studies involving online interventions without face-to-face contact (HMRF 15161231 & 19181152). Ethics approval will be obtained from the Survey and Behavioural Research Ethics Committee of the Chinese University of Hong Kong and the joint CUHK-NTEC Clinical Research Ethics Committee.

Official data showed that smartphone ownership among residents aged ≥60 years was 65% in 2017. In a previous survey, we successfully recruited 750 community-dwelling elderly through random telephone sampling within 3 months (10). Majority of them had a smartphone (73.7%), and were willing to receive PV promotion via smartphone (76.6% among those who had smartphone). It is hence feasible to recruit 374 participants within 6 months.

Randomization and baseline survey

Participants will be interviewed to record background characteristics and potential confounders after randomization at Baseline. At the end of baseline survey, the interviewers will have their WhatsApp connected to the Chatbot, and provide instructions on how to use Chatbot. A randomization algorithm is built in the Chatbot. The Chatbot will randomly allocate participants evenly either to the intervention group or to the control group. The automated randomization process takes place online and is concealed from the research team. The Chatbot will automatically run a training module guiding users to use its functions in an elderly-friendly way when users access it for the first time.

Health promotion for the intervention group

<u>Overview</u>: The Chatbot will deliver intervention tailored to participants' SOC regarding PV uptake in an interactive way in four monthly sessions through WhatsApp.

<u>Intervention at Month 0, 1, 2 and 3</u>:

The Chatbot will guide the conversation as below:

1) Initiate the conversation: The Chatbot greets the participants and states the topics it covers.

2) Identify SOC: The Chatbot will ask participants two simple questions (whether he/she intends to take up PV in the next year, and whether he/she plans to do so in the next month). Participant can verbally answer the question or choose a response shown on the screen. The Chatbot verbally confirms the response to avoid misunderstanding. Pre-contemplation stage is defined as not intending to take up PV in the next year, contemplation stage is defined as intending to take up PV in the next year but without plans to do so in the next month, while preparation stage is defined as having plans to take up PV in the next month.

3) Deliver interventions tailored to SOC

The interventions will modify perceptions that were associated with PV uptake in our groundwork (perceived risk of PD/IPD, perceived benefits and barriers of PV uptake, suggestion made by significant others, self-efficacy).

i) Pre-contemplation stage: the health communication messages will increase participants' awareness about the importance of PV uptake. It covers: a) information about high risk of PD/IPD among elderly, b) PV is effective to protect elderly and their family member, c) local health authorities/physicians strongly recommend elderly to take up PV, and receiving COVID-19 vaccination will not affect PV, and d) free PV is available for you at public clinics.

ii) Contemplation stage: the health communication messages will:

a) Increase perceived pros: The Chatbot emphasizes the promising efficacies of PV in preventing PD and IPD, and protect family members through herd immunization.
b) Reduce perceived cons: The Chatbot explains common side-effects of PV are mild and severe side-effects are rare. Testimonial video of vaccinated elderly peers regarding side-effects will be played. Our groundwork showed that elderly often perceived peers' experience to be credible. In addition, the Chatbot emphasizes PV uptake is free and convenient in a nearby public clinic to reduce concerns related to cost and inconvenience.
c) Provide suggestion and encourage to make specific plan: The Chatbot emphasizes that PV is strongly recommended by local health authorities to elderly, and encourages them to make plan to receive PV.

iii) Preparation stage: the Chatbot assists participants to develop and implement concrete action plan to increase their perceived self-efficacy related to PV. The contents will cover: a) location, working hours and contact of public hospitals/clinics offering free PV for elderly near where they are living, and b) ask participants when, where and how they would make an appointment to receive PV. Participants are reminded that they should keep sufficient interval (at least 2 weeks) between PV and COVID-19 vaccination. Participants could provide a response verbally or in written text. RCT showed that asking people to provide where, when, and how they want to perform a behaviour could increase their self-efficacy (24). The Chatbot will document their plans, and automatically send three weekly messages reminding the participants to carry out their plans to reinforce their self-efficacy.

Visual graphs and Chinese subtitle are shown on the screen during the conversation. The

Chatbot will document prior conversations with the users and avoid giving a repetitive dialogue.

5) Address concerns related to PV to reduce perceived cons related to PV

Participants can select one or more concerns that applied to them from a list of common concerns related to PV identified by our groundwork and focus group of elderly. Addressing concerns that are perceived to be important increased efficacy of the intervention (20). Participants can also raise their concern which is not in the list verbally or in written text. The Chatbot will retrieve relevant information from the knowledge graph and prepare a response. If there is no further enquiry, the Chatbot will remind them about the time for next conversation and end the dialogue.

Starting from Month 1, the Chatbot will first ask whether the participant has taken up PV. For those who have done so, the Chatbot will make a record and terminate the program automatically. For those who have not done so, the Chatbot will identify their SOC, deliver stage-tailored interventions and address their concerns related to PV.

Health promotion for the control group

The Chatbot will automatically send a standard online intervention covering the same key information as those for the intervention group at Month 0, 1, 2, & 3. The contents will include: 1) high risk of PD/IPD among older adults, 2) efficacy and safety of PV, and 3) free PV is available for older adults at public clinics. We will also prepare different versions of the interventions, and the Chatbot will randomly select one version for the participants in each session to prevent participants from receiving a same intervention twice.

During the intervention period, both groups can contact our project staff through the hotline. Personal information such as participants' telephone number and names (optional) will be stored in the Chatbot server during the intervention period and are protected by a password. Only the principal investigator has access to the data. Such data will be deleted from the Chatbot server after the intervention period.

Development and maintenance of the Chatbot

A fully-automated AI-Chatbot will be developed in collaboration with IT engineers on the WhatsApp platform for their wide popularity and user-friendly interface for extensions. The knowledge graph will be prepared by focus groups of 30 elderly and interview of our Co-Is and is evolving over time with increasing amount of prior conversations. The Chatbot will use a mixture of techniques so that a dialogue will not merely be rule-based, but it can also learn from the evolving knowledge graph through machine learning. We will pilot the Chatbot among another 30 elderly to improve its accuracy of human-machine interactions. The Chatbot will keep learning from the evolving knowledge graph through machine learning during the intervention period. These measures allow the Chatbot have adequate accuracy and consistency in delivering online intervention according to the protocol, and avoid the Chatbot going repetitive and off-topic responses after extended usage. The same professional team will

maintain the healthy state of the Chatbot. They will review its performance every month. A feedback loop is incorporated in the Chatbot, users are invited to leave commentary. Improvements will be made accordingly.

Measures to avoid contamination and reduce drop-out rate

First, the participants cannot download the conversation with Chatbot or message sent by the Chatbot. Second, only one member per household will be recruited, the within-household contamination is hence avoided. Third, since the recruitment is based on population-based random telephone contacts, the chance that two participants knowing each other and sharing the intervention materials is extremely rare and not of practical concern. The PV program is implemented all year round in Hong Kong. The seasonal effects on PV program are limited. Moreover, the PV program is not affected by the ongoing massive COVID-19 vaccination program. The arrangement of receiving a PV remains the same as the period before COVID-19. According to the interim guidance of COVID-19 vaccination in Hong Kong, administration of COVID-19 vaccination should be 14 days before or after another prophylactic vaccine (including PV) to allow clearer ascertainment of potential adverse effects. It is possible that participants who plan to receive COVID-19 vaccination would have concerns related to the administration of PV. Additional health communication messages on: 1) receiving COVID-19 vaccination would not affect PV and 2) one should keep sufficient interval (at least 2 weeks) between COVID-19 vaccination and PV are provided to participants in both groups to reduce potential interference caused by COVID-19 vaccination program.

Measurements

Primary outcome: Prevalence of self-reported PV uptake at T1 is the primary outcome. This outcome will be validated by requesting participants to send us an image of the receipt, hiding personal identification and via the same WeChat/WhatsApp account used in this project. No incentive is offered for validating PV uptake. Weekly reminders will be sent to them if they fail to do so. Same verification procedure for vaccination uptake has been used in our HMRF project (13141651 & 19181152).

Secondary outcomes:

1) SOC related to PV uptake measured at T1.

2) Engagement with the Chatbot. The number of intervention sessions received and completed by all participants and number of questions asked by the participants in the intervention group will be retrieved from the Chatbot system. The participants' subjective experience related to behavioural, cognitive and affective engagement with the Chatbot will also be measured.

Incentives for the surveys

A supermarket coupon (HK$50) will be mailed to an address provided by the participant upon completing the T0 and T1 follow-up surveys to ensure response rate and as an appreciation for their time spent. Same amount of incentive for completing baseline/follow-up surveys was used in RCT targeting elderly in Hong Kong (HMRF 15161231 & 19181152).

Pilot study

A pilot study of six eligible participants randomized into the two groups will be conducted to test the logistics of the intervention. Refinements will be made if necessary.

Sample size planning

Our pilot data showed that 18.6% of the participants intended to take up free PV at public hospitals/clinics in the next year after being briefed about some facts of PV. Meta-analysis showed that 43-62% of those with a behavioural intention would translate it into action. For planning purposes, we conservatively assume 30% of those in the control group would show an intention after exposure to the interventions, and 50% of those with such an intention would take up PV (15% of the control group). For planning purpose, we use smallest detectable difference of 15% between the intervention group and the control group (30% in the intervention group). We need 121 per group to achieve planned effect sizes and power of 0.8 and alpha of 0.05. Assume that the loss-to-follow-up rate will be 35% at T1, a total of 187 participants per group will be required. The total sample size will be 374 (PASS 11.0; NCSS; Kaysville; U.S.).

(iii) <u>Study design</u> A non-blinded 2-arm parallel RCT will be conducted. Participants will be interviewed by phone at T0 and T1. Participants present at T1 will be asked a final evaluation questionnaire recording uptake of PV during the follow-up period and the secondary outcomes.

(iv) <u>Data processing and analysis</u> Intention-to-treat analysis (ITT) will be performed. Assuming the data are missing at random, a Markov Chain Monte Carlo Method will be used to impute missing primary and secondary outcomes. Variables used to impute the missing values of these outcomes include participants' background characteristics and baseline values of these outcomes. Chi-square tests will be used to inspect between-group balances of baseline characteristics. The relative risk reduction, absolute risk reduction, and number need to treat and their 95% confidence interval will be calculated by using Excel. Logistic regression (for binary variables) and linear regression models (for continuous variables) will be used to test the between-group difference in the primary and the secondary outcomes, after controlling for any baseline confounders with $p<.2$ in between-group comparisons.

e) Impact on People's Health and Health Services as well as Plan to Disseminate Research Findings to End Users:

The underlying purpose of the study is to prevent community-acquired pneumonia, invasive pneumococcal diseases (IPD), and secondary bacterial pneumonia by promoting uptake of pneumococcal vaccination (PV) among community dwelling elderly aged ≥65 years. It can also reduce mortality and morbidity caused by secondary and co-infection of bacterial pneumonia following seasonal influenza. In Hong Kong, pneumonia/IPD is a serious public health threat for elderly. Although PV is recommended and provided for free to elderly in Hong Kong, the coverage of PV in this group was very low (<20%). International experiences showed that PV uptake among elderly in countries where it is free and recommended but without effective interventions might be low.

The proposed intervention has strong applications to infectious diseases control in Hong Kong. PV is an

efficacious public health tool to prevent elderly from pneumonia/IPD. Although efforts promoting PV among elderly existed both locally and internationally, effective and sustainable interventions are greatly needed but lacked. We will develop an online intervention based on our groundwork and the Stage of Change. We will compare the efficacy of such intervention with the control group by using randomized controlled trial, which is the strictest evaluation method giving evidence of the highest quality. Our intervention is tailored to participants' stage of change of taking up PV, which could increase its efficacy. All intervention will be delivered by a fully-automated Chatbot that can process natural spoken language (Cantonese), which is especially suitable for elderly users. Making use of Chatbot can also enhance compliance with the online interventions. It can initiate personalized interventions with the participants regarding PV uptake. Since the Chatbot has virtually no maintenance cost, it is especially suitable to deliver our proposed intervention. The sustainability of our intervention is expected to be high. The intervention, if found to be significant and translated into service, would increase coverage of PV among elderly and enhance effectiveness of the governmental PV programs.

We will take the following steps to disseminate research findings to end users. First, we will work with 3-5 non-governmental organizations (NGO) (e.g., Hong Kong Council of Social Service, the Hong Kong Society of the Aged, etc.) and governmental sectors (e.g., District Health Centres) providing services to older adults in Hong Kong to conduct an implementation study. The aims are to identify and overcome barriers of implementing Chatbot-based PV promotion in real-world settings and to facilitate adoption of such promotion by these NGO and District Health Centres. Through health seminars/webinars/conferences disseminating relevant findings, we will try to integrate Chatbot-based PV promotion as a part of rountine services in a larger number of NGO (26 in total) and District Health Centres/District Health Centre Express (18 in total) in long term. It would increase coverage of the health promotion and PV coverage, and hence reduce related disease burden at population level.

f) Key References:

1. Varon E, Mainardi JL, Gutmann L. Streptococcus pneumoniae: still a major pathogen. Clinical microbiology and infection : the official publication of the European Society of Clinical Microbiology and Infectious Diseases. 2010;16(5):401.

2. Department of Health. Health Facts of Hong Kong 2019 Edition. Available at: https://www.dh.gov.hk/english/statistics/statistics_hs/files/Health_Statistics_pamphlet_E.pdf. 2019.

3. Center for Disease Control and Prevention. Pneumococcal Diseases: Surveillance and Reporting. Available at: https://www.cdc.gov/pneumococcal/surveillance.html. 2016.

4. Centre for Health Protection. Report on IPD. Available at: https://www.chp.gov.hk/en/resources/29/636.html. 2019.

5. Centre for Health Protection. Updated Recommendations on the Use of Pneumococcal Vaccines for High-risk Individuals. Available at: https://www.chp.gov.hk/files/pdf/updated_recommendations_on_the_use_of_pneumococcal_vaccines_amended_120116_clean_2.pdf. 2016.

6. Morris DE, Cleary DW, Clarke SC. Secondary Bacterial Infections Associated with Influenza Pandemics. Frontiers in microbiology. 2017;8:1041.

7. Bonten MJ, Huijts SM, Bolkenbaas M, Webber C, Patterson S, Gault S, et al. Polysaccharide

conjugate vaccine against pneumococcal pneumonia in adults. The New England journal of medicine. 2015;372(12):1114-25.

8. Gilchrist SA, Nanni A, Levine O. Benefits and effectiveness of administering pneumococcal polysaccharide vaccine with seasonal influenza vaccine: an approach for policymakers. American journal of public health. 2012;102(4):596-605.

9. La EM, Trantham L, Kurosky SK, Odom D, Aris E, Hogea C. An analysis of factors associated with influenza, pneumoccocal, Tdap, and herpes zoster vaccine uptake in the US adult population and corresponding inter-state variability. Human vaccines & immunotherapeutics. 2017:1-12.

10. Janz NK, Becker MH. The Health Belief Model: a decade later. Health Education Quarterly. 1984;11(1):1-47. Retrieved from http://heb.sagepub.com/content/11/1/1.short.

11. Prochaska JO, Velicer WF. The transtheoretical model of health behavior change. American journal of health promotion : AJHP. 1997;12(1):38-48.

12. Noar SM, Benac CN, Harris MS. Does tailoring matter? Meta-analytic review of tailored print health behavior change interventions. Psychological bulletin. 2007;133(4):673-93.

13. Lach HW, Everard KM, Highstein G, Brownson CA. Application of the transtheoretical model to health education for older adults. Health promotion practice. 2004;5(1):88-93.

14. Paiva AL, Lipschitz JM, Fernandez AC, Redding CA, Prochaska JO. Evaluation of the acceptability and feasibility of a computer-tailored intervention to increase human papillomavirus vaccination among young adult women. Journal of American college health : J of ACH. 2014;62(1):32-8.

15. Redding CA, Brown-Peterside P, Noar SM, Rossi JS, Koblin BA. One session of TTM-tailored condom use feedback: a pilot study among at-risk women in the Bronx. AIDS Care. 2011;23(1):10-5.

16. Chen H, Liu, X., Yin, D., Tang, J. A survey on dialogue systems: Recent advances and new frontiers. Acm Sigkdd Explorations Newsletter. 2017;19(2):25-35.

17. Fadhil A, Gabrielli S. Addressing challenges in promoting healthy lifestyles: the al-chatbot approach. PervasiveHealth '17: Proceedings of the 11th EAI International Conference on Pervasive Computing Technologies for Healthcare May 2017 https://doi.org/10.1145/3154862.3154914. 2017:261-5.

18. Pereira J, Díaz Ó. Using Health Chatbots for Behavior Change: A Mapping Study. Journal of medical systems. 2019;43(5):135.

19. Wang H, Zhang QP, Ip M, Lau JT. Social media-based conversational agents for health management and interventions. IEEE. 2018;51(8):26-33.

20. Pereira J, Diaz O. Using Health Chatbots for Behavior Change: A Mapping Study. Journal of medical systems. 2019;43(5):135.

21. Muellmann S, Forberger S, Mollers T, Broring E, Zeeb H, Pischke CR. Effectiveness of eHealth interventions for the promotion of physical activity in older adults: A systematic review. Preventive medicine. 2018;108:93-110.

22. Kwan RY, Lee D, Lee PH, Tse M, Cheung DS, Thiamwong L, et al. Effects of an mHealth Brisk Walking Intervention on Increasing Physical Activity in Older People With Cognitive Frailty: Pilot Randomized Controlled Trial. JMIR mHealth and uHealth. 2020;8(7):e16596.

23. King AC, Bickmore TW, Campero MI, Pruitt LA, Yin JL. Employing virtual advisors in preventive

care for underserved communities: results from the COMPASS study. Journal of health communication. 2013;18(12):1449-64.

24. Milkman KL, Beshears J, Choi JJ, Laibson D, Madrian BC. Using implementation intentions prompts to enhance influenza vaccination rates. Proceedings of the National Academy of Sciences of the United States of America. 2011;108(26):10415-20.
