# Supplementary material for: A Hybrid Chatbot to Promote Pneumococcal Vaccination Among Older Adults: A Randomized Clinical Trial
Source: JAMA Netw Open. 2025 Oct 8;8(10):e2535813. doi: 10.1001/jamanetworkopen.2025.35813 (PMC12509012; doi:10.1001/jamanetworkopen.2025.35813)
Supplement: Supplement 3. — Data Sharing Statement [file jamanetwopen-e2535813-s003.pdf]

Data Sharing Statement

Wang. A Hybrid Chatbot to Promote Pneumococcal Vaccination Among Older Adults. *JAMA Netw Open*. Published October 08, 2025. doi:10.1001/jamanetworkopen.2025.35813

Data
Additional Information: Trial registration: ClinicalTrial.gov identifier: NCT05772117
Data available: No

Additional Information
Explanation for why data not available: The datasets generated and/or analyzed during the current study are not publicly available as they contain sensitive personal behaviors but are available from the corresponding author on reasonable request.
